# TXNIP/TBP-2: A Master Regulator for Glucose Homeostasis

**DOI:** 10.3390/antiox9080765

**Published:** 2020-08-18

**Authors:** Eiji Yoshihara

**Affiliations:** 1 The Lundquist Institute for Biomedical Innovation at Harbor-UCLA Medical Center, Torrance, CA 90502, USA; eiji.yoshihara@lundquist.org; 2 David Geffen School of Medicine at UCLA, Los Angeles, CA 90095, USA

**Keywords:** TXNIP, glucose homeostasis, TBP-2

## Abstract

Identification of thioredoxin binding protein-2 (TBP-2), which is currently known as thioredoxin interacting protein (TXNIP), as an important binding partner for thioredoxin (TRX) revealed that an evolutionarily conserved reduction-oxidation (redox) signal complex plays an important role for pathophysiology. Due to the reducing activity of TRX, the TRX/TXNIP signal complex has been shown to be an important regulator for redox-related signal transduction in many types of cells in various species. In addition to its role in redox-dependent regulation, TXNIP has cellular functions that are performed in a redox-independent manner, which largely rely on their scaffolding function as an ancestral α-Arrestin family. Both the redox-dependent and -independent TXNIP functions serve as regulatory pathways in glucose metabolism. This review highlights the key advances in understanding TXNIP function as a master regulator for whole-body glucose homeostasis. The potential for therapeutic advantages of targeting TXNIP in diabetes and the future direction of the study are also discussed.

## 1. Introduction

**Figure 1:**
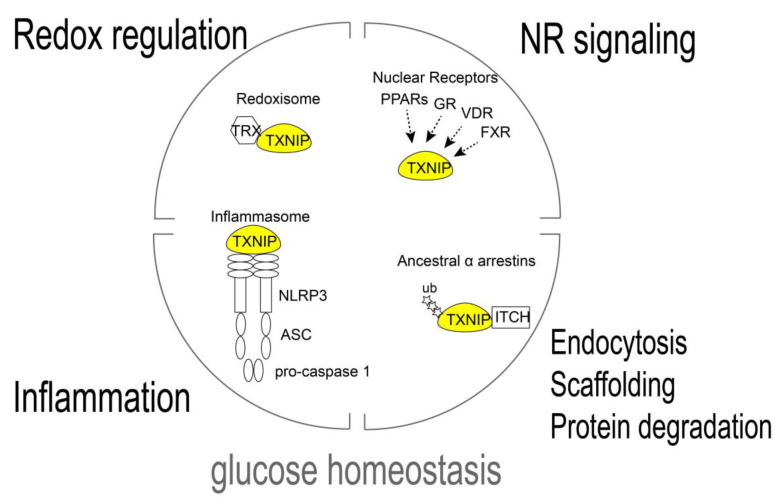
TXNIP regulates glucose homeostasis as signal complex. The TRX/TXNIP signal complex, redoxisome, is the basis of TXNIP regulation of the reduction–oxidation (redox) response. TXNIP has been known to bind NOD-like receptor protein 3 (NLRP3) and activate the inflammasome. TXNIP is a member of the ancestral α-Arrestin family and TXNIP binds to the Itchy E3 Ubiquitin Protein Ligase (ITCH) and facilitates the ubiquitination of the substrates. TXNIP is transcriptionally regulated by nuclear receptors (NRs) such as peroxisome-proliferator activated receptors (PPARs), glucocorticoid receptor (GR), vitamin D receptor (VDR), and farnesoid X receptor (FXR) in a cell type specific manner. These signal complex/transducers are involved in the physiological functions of TXNIP, including the regulation of glucose homeostasis.

Thioredoxin interacting protein (TXNIP) was first cloned as an up-regulated gene by 1,25-dihydroxy D3 (Vitamin D3) and named as Vitamin D3 upregulated protein 1 (VDUP1) in the human leukemia cell line (HL-60) derived from a patient with promyelocytic leukemia [1]. Subsequentially, TXNIP was identified as a thioredoxin (TRX) binding protein by yeast two-hybrid assay and named thioredoxin binding protein-2 (TBP-2^)^ [2]. Later studies identified a TBP-2 nonsense mutation gene in HcB-19 mice that confers the feature of familial combined hyperlipidemia (FCHL), and VDUP1/TBP-2 was re-named TXNIP [3]. TXNIP was shown to lessen the reducing activity of TRX in an HL-60 cell line. Although a later study revealed no clear evidence of the vitamin D3 responsive element (VDRE) in the promoter of TXNIP and regulation of TXNIP expression by Vitamin D3 in other cell types is limited [4], the direct protein–protein binding of TXNIP to TRX as well as the responsiveness to extracellular stimulation makes it an interesting target for this study. The concept of the TRX–TXNIP complex acting as the redox sensitive signal transducer “redoxisome” was previously discussed [5]. The fundamental function of TXNIP is to act as the oxidative stress responsive signal transducer redoxisome, which explains the wide influence of its biological function. In addition to nuclear receptor (NR) signaling and redox regulation, physical binding of TXNIP to the NOD-like receptor protein 3 (NLRP3) inflammasome or E3-ubiquitin ligases sheds light on TXNIP function Figure 1. In this review, we summarize the decades of work analyzing TXNIP as a master regulator of glucose homeostasis by integrating its function, physiological role, and diabetes development.

## 2. TXNIP/TBP-2 in Whole-Body Glucose–Lipid Metabolic Regulation

**Figure 2:**
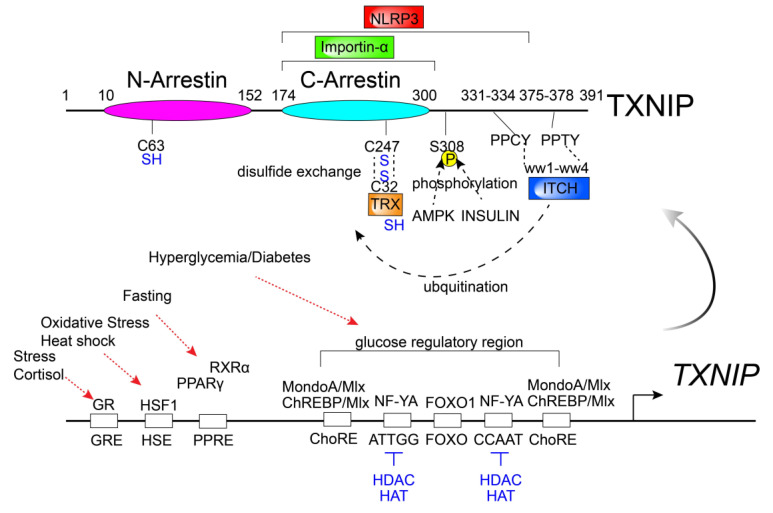
Transcriptional and post-translational modification and protein interacting domains of TXNIP. TXNIP gene expression is regulated by various stimuli. Post-translational modifications and protein interactions of TXNIP influence its protein stability, localization and function in a cellular and context-dependent manner. Protein scaffolding and the biological function of TXNIP are biased in the C-arrestin domain. Two PPxY motifs (PPCY and PPTY) of Txnip bind to the four WW domains of E3 ubiquitin ligase, ITCH. Insulin and AMPK facilitate the protein degradation by serine 308 (S308) phosphorylation of TXNIP. TXNIP forms disulfide bonds with reduced TRX by disulfide exchange through its cysteine 247 (C247). Glucocorticoid–glucocorticoid responsive element (GR–GRE), heat shock factor 1–heat shock element (HSF1–HSE), peroxisome proliferator-activated receptor γ/retinoid X receptor α–peroxisome proliferator-activated receptor element (PPARγ/RXRα–PPRE), MLX interacting protein/Max-like protein X (MondoA/Mlx – ChoRE) or carbohydrate-responsive element-binding protein/Max-like protein X–carbohydrate response element (ChREBP/Mlx-ChoRE), nuclear transcription factor Y subunit α–CCAAT motif (NF-YA–ATTGG/CCAAT), Forkhead Box O1 (FOXO1)–putative FOXO1 binding site (FOXO).

TXNIP has emerged as a master regulator for glucose and lipid metabolism, largely because of the finding of metabolic disordering phenotypes in TXNIP mutants or gene knockout (KO) mice. Earlier discovery of the nonsense mutation of TXNIP in HcB-19 mice revealed the important physiological function of TXNIP in glucose–lipid homeostasis [3,6]. HcB-19 mice were identified as a variant of the C3H strain, which exhibits a phenotype similar to familial combined hyperlipidemia (FCHL), such as elevated levels of plasma triglyceride, cholesterol, and free fatty acids [3,6]. In addition, HcB-19 mice have an abnormal glucose metabolic phenotype, including hyperinsulinemia, hypoglycemia, and ketosis during fasting [7,8,9]. Later studies revealed that many metabolic phenotypes identified in HcB-19 are reproducible in whole-body TXNIP knockout mice (TXNIP-WKO). With a genetic targeting strategy, we previously reported that TXNIP-WKO causes predisposition to death with hypoglycemia, hyperinsulinemia, ketosis, and abnormal liver steatosis during fasting [10,11]. Transcriptional dysregulation of feeding-fasting transition was observed in the liver of TXNIP-WKO, such as upregulation of the “fasting” signal, peroxisome proliferator activated receptor-alpha (PPAR-α), in the “feeding” status and upregulation of the feeding signal, sterol response element binding protein (SREBP), in the fasting status. Interestingly, genetic deletion or mutation of TXNIP in diabetic model mice such as ob/ob mice [12,13], STZ-induced diabetic [13,14] mice and HFD [15,16] mice exhibited improved glucose tolerance and remission of hyperglycemia. In addition, the correlation of the human insulin/glucose clamp study revealed that inversed correlation of TXNIP expression and insulin dependent glucose uptake in skeletal muscle and TXNIP expression are associated with the risk of the pathogenesis of type 2 diabetes (T2D) in humans [17]. These findings led to the idea that suppression of TXNIP in the prediabetic and diabetic conditions may be beneficial for treating human diabetes. TXNIP expression is sharply regulated in many tissues by physiological conditions, which limits the glucose metabolism such as fasting [11] and obesity-prediabetic condition [12]. Although cell type specificity of TXNIP expression has been observed, nutrients sensors, including (NRs) such as peroxisome proliferator activated receptors (PPARs) [11,18], vitamin D receptor (VDR) [1,2,18], and glucocorticoid receptor (GR) [19,20,21], also upregulate TXNIP transcriptional level and AMPK downregulates TXNIP by promoting protein degradation [22]. Glucose regulates TXNIP expression through transcriptional factor carbohydrate-response element-binding protein (ChREBP) in liver and β cells [23,24] and through MondoA/Mlx in skeletal muscle and the heart [25]. Insulin reciprocally suppresses TXNIP expression through insulin signaling in diverse tissues [11,12,13,16,26,27,28,29,30,31,32,33]. TXNIP is induced by hypoxic conditions by hypoxia-inducible factor 1 α (HIF-1α) in endothelial cells and various cancer cells [34,35,36,37,38,39,40,41]. HIF-1α is an important regulator of glycolysis, therefore local glucose metabolism may also be influenced by the HIF1-α/TXNIP axis under hypoxic conditions [42]. These findings suggest that the dynamic change of TXNIP expression is important to regulate nutritional and hormone sensing metabolic regulation Figure 2. Although the mutation or deletion of TXNIP shows that the imbalance of redox regulation, such as sulhydryl-redox or PTEN disulfide reduction, influences metabolic impairment in vivo [16,43], redox-independent, tissue specific TXNIP functions in metabolic regulation have also been widely observed. The specific role of TXNIP in metabolic tissues is discussed below.

### 2.1. TXNIP/TBP-2 in Immune Cells

TXNIP plays an important role in various types of immune cells. It was found that the number of Natural Killer (NK) cells is profoundly reduced in TXNIP-WKO, which was linked to poor ability of tumor rejection in TXNIP-WKO [44]. We previously reported that dendritic cells (DCs) derived from TXNIP are defective in inducing T-cell responses [45]. Although these results suggest that TXNIP is required for the maintenance of immune cell function in normal physiological conditions, in the tumorigenic condition, TXNIP expression shows cytotoxic effects in tumorigenic immune cells. For example, TXNIP expression is inversely correlated with hematopoietic malignancies, such as adult T-cell leukemia (ATL), and its responsiveness to glucocorticoids to induce apoptosis in T-cell lines infected with human T lymphotropic virus type-I (HTLV-I), the causative virus of adult T cell leukemia (ATL) [20,21]. It is still poorly understood how these TXNIP functions in immune cells affect metabolic phenotypes in TXNIP-WKO, while interestingly, we have previously found that lipopolysaccharide (LPS) injection in TXNIP-WKO unexpectedly exhibited dysregulation of the lipid and glucose metabolisms, such as hyperinsulinemia, hypoglycemia, fat deposition in the liver and kidney, organ injuries, glycogen depletion, and elevation of serum lipid derivatives such as free fatty acids, triglycerides, and cholesterol [46,47]. Glucose supplementation extended the survival in TXNIP-WKO under LPS challenge. These results suggest that hypoglycemia promoted by hyperinsulinemia may be a critical risk factor for mortality in circumstances in which fatty acid utilization is impaired during endotoxemia in TXNIP-WKO [46]. Notably, these metabolic disorder phenotypes resemble the phenotypes which TXNIP-WKO exhibit under fasting conditions [10,46]. These results suggest that defective immune response may contribute to the defect of metabolic regulation in TXNIP-WKO.

A remarkable feature of TXNIP in inflammatory signaling is the physical binding with NLRP3, a central component of inflammasome (Figure 2) [48]. The inflammasome activators, such as uric acid crystals, induce the dissociation of TXNIP from TRX in a reactive oxygen species (ROS)-sensitive manner and lead to the binding of NLRP3. TXNIP deficiency impaired the activation of the NLRP3 inflammasome and the subsequent secretion of interleukin-1β (IL-1β) in macrophages. Although TXNIP-NLRP3 inflammasome axis is independent with known NLRP3 activation by oligomers of islet amyloid polypeptides (IAPP) [49] (a protein that forms amyloid deposits that has been observed during type-2 diabetes in pancreatic β cells), TXNIP-NLRP3 inflammasome axis seems to be an important regulator for specific tissue inflammation in redox dependent and independent manners [48,50,51,52,53,54,55,56,57,58,59,60,61,62,63,64,65,66,67,68,69,70,71].

### 2.2. TXNIP/TBP-2 in Pancreatic Islets

TXNIP is one of the genes most highly upregulated by high glucose stimulation in murine and human β cells [72,73]. Glucose sensing TXNIP induction is tissue specific, and in the case of β cells, the glucose sensor carbohydrate-response element-binding protein (ChREBP) directly binds to the promoter region of TXNIP and enhances the gene expression [72]. ChREBP is regulated by glucose metabolites such as glucose-6 phosphate (G6P), xylulose 5-phosphate (X5P), and fructose 2,6-biphosphate. The major regulator of glycolysis has been implicated to bind the glucose-response activation conserved element (GRACE) of ChREBP to activate cytosolic–nuclear translocation for further downstream regulation of β cell function including β cell proliferation/compensation and death [74]. Glucose responsiveness of TXNIP is linked to the high glucose-induced apoptosis induction. One of notable function of TXNIP in β cells is the induction of apoptosis in response to high glucose [72,75]. TXNIP is induced by various kinds of stimulation and links to the apoptosis induction by streptozotocin [14,76], ER-stress [77,78,79], dexamethasone/glucocorticoid [80], lipids [81], inflammation/cytokines [14,82], and oxidative stress [48,83]. The stress-induced upregulation of TXNIP is observed in the pancreatic islets during the progression of diabetes in both mice [12] and humans [17]. TXNIP deficiency is protective for β cells from mouse models of both type-1 (T1D) and type-2 diabetes (T2D) [12,13,77]. Mechanistically, TXNIP inhibits TRX (in the cytosol, nucleus) and thioredoxin-2 (TRX2, mitochondria) and enhances oxidative stresses. TXNIP is predominantly expressed in the cytosol and nucleus, while upon oxidative stress TXNIP shuttles to the mitochondria and interacts with TRX2 and releases the interaction between TRX2 and apoptosis signal regulating 1 protein (ASK1), which leads to the activation of ASK1 to induce β cell apoptosis [84,85]. TXNIP induces several microRNAs (miRNAs) to promote β cell apoptosis. TXNIP increases expression of pro-apoptotic miR-200, which inhibit zinc finger E-box-binding homeobox 1 (Zeb1) to promote apoptosis [86]. Although TXNIP undoubtedly controls β cell’s apoptosis, induction of apoptosis by TXNIP typically takes from 24 h to a few days by TXNIP overexpression [12]. Since the high glucose response TXNIP expression is rapid, TXNIP functions other than apoptotic regulation may be crucial for β cell function. TXNIP deficiency enhances glucose-stimulated insulin secretion (GSIS) under feeding-fasting nutritional regulation [11]. TXNIP deletion improves GSIS function in the T2D model ob/ob mice, which contributes the amelioration of hyperglycemia [12]. These results suggest that prior to the apoptotic induction, TXNIP acts as suppressor of GSIS, which may save glucose utilization under the fasting and/or stress induced conditions. In addition to the physiological regulation of insulin secretion and apoptosis, it has been reported that TXNIP mediates miR-204 induction and directly inhibits INSULIN transcription through down regulation of MAFA [87]. TXNIP has also been shown to mediate glucose dependent upregulation of islet amyloid polypeptide (IAPP) through miR-124a [88]. IAPP upregulation has been suggested as a marker for functional maturation during human β cells development [89]. Accumulation of IAPP is known to promote inflammation and β cell dysfunction [49]. These important gene regulatory functions of TXNIP have just started to be revealed. TXNIP may have differential functions under stress or healthy physiological conditions.

### 2.3. TXNIP/TBP-2 in Peripheral Tissues (Muscle, Adipose, Liver)

Regulation of glucose metabolism by TXNIP in muscle is highlighted by the human glucose/insulin physiological clamp study, which showed the dynamic regulation of TXNIP by glucose and insulin [17]. In addition, TXNIP expression is inversely correlated with glucose uptake in healthy humans and it is upregulated in skeletal muscle of prediabetic and diabetic T2D patients [17]. TXNIP expression in muscle cells, including vascular smooth muscle, skeletal muscle, and cardiomyocytes, is known to be regulated by MAPK signaling and PI3K/insulin signaling. Early studies identified that glucose-induced TXNIP expression is abolished by the P38 MAPK inhibitor PD169316 [43]. In addition, glucose-induced TXNIP expression is upregulated by phosphoinositide 3-kinase (PI3K) inhibitor wortmanin but not the ERK inhibitor U0126, Gi inhibition by the pertussis toxin, or protein kinase C (PKC) inhibition by GF109203X in human aortic smooth muscle cells (SMCs) [43]. PDGF-BB enhanced phosphorylation of PI3K/AKT in the central network of insulin signaling and reduced TXNIP transcription in both low and high glucose conditions in SMCs [43]. Serine 308 of TXNIP was shown to be targeted for the phosphorylation induced by insulin [32] or AMPK [22], which lead to the rapid degradation of TXNIP protein (Figure 2). TXNIP induction by glucose is regulated by MondoA, an analog of ChREBP, at a transcriptional level in skeletal muscle and the heart [25]. It has been reported that the mammalian target of rapamycin (mTOR), a downstream molecule of PI3K, physically binds to MondoA in the cytoplasm and prevents MondoA–Mlx complex formation and restricts MondoA’s nuclear entry and reduces transcriptional activation of TXNIP [25,90]. Overexpression of TXNIP suppresses glucose uptake while knockdown or knockout of TXNIP enhances glucose uptake in skeletal muscle and adipose in vitro and in vivo [12,16,17,32,91]. The amount of phosphatidylinositol (3,4,5)-trisphosphate (PIP3) is regulated by the balance of PI3K and the phosphatidylinositol 3-phosphatase PTEN [92]. Oxidation of a small fraction of critical cysteine residues is regulated by TRX and inactivates PTEN; therefore, redox signaling influences insulin and growth factor receptor signaling via expansion of PIP3 accumulation, which activates the downstream signaling kinase Akt [93,94,95,96,97]. It has been shown that TXNIP regulates PTEN disulfide reduction via TRX and glucose uptake [16]. We have shown that TXNIP preserves almost full insulin sensitivity by increasing insulin responsiveness of AKT phosphorylation under severe insulin resistance conditions, such as ob/ob mice [12]. Regulation of whole-body insulin sensitivity by TXNIP is modulated in white adipose tissue. Larger adipose-size phenotypes in both TXNIP-WKO and adipose-specific TXNIP deletion (TXNIP-AKO) in control treatments and high-fat/diabetic conditions were observed, and this phenotype displayed larger capacity for the storage of nutrients, including glucose, compared wild type (WT) mice [12,15]. TXNIP also controls hepatic gluconeogenesis [98,99]. TXNIP-WKO exhibited abnormal liver steatosis and impaired gluconeogenesis during fasting, which may contribute the phenotype of hypoglycemia-induced predisposition for death [10,11]. Similarly, liver specific TXNIP deletion (TXNIP-LKO) caused hypoglycemia and ketoacidosis, which is consistent with the finding of poor glucose production and increased β-hydroxybutyrate release from isolated hepatocytes from TXNIP-LKO mice [99]. Forkhead box protein O1 (FOXO1), a master transcriptional factor for gluconeogenesis in the liver, directly regulates TXNIP expression through the binding of the promoter region of TXNIP which contains a conserved consensus sequence, ′GTAAACAA′, of the FOXO binding site [100,101]. Taken together, these early discoveries revealed the tight link between TXNIP expression and glucose or insulin signaling in peripheral tissues.

### 2.4. TXNIP/TBP-2 in Central Nervous System

Although the direct action of the hormones in peripheral tissues is sufficient to mediate the regulation of glucose/nutritional handling, the crucial role of the central nervous system (CNS) in glucose homeostasis [102] has been widely acknowledged. The mediobasal hypothalamic (MBH) TXNIP expression is changed depending on the nutritional condition. Fasting induces TXNIP expression, whereas refeeding, leptin infusion, or insulin infusion reduces TXNIP expression in the hypothalamus [103]. Experimental diabetes models induced by STZ, or polygenic models of obesity, adult onset T2D, diet-induced hyperglycemia and obesity, and NONcNZO10/LtJ mice all have MBH TXNIP expression increased at feeding status, suggesting that TXNIP is responsible for the nutritional sensing and pathophysiological conditions in MBH [103]. TXNIP overexpression but not C247S-mutated (binding site with TRX) TXNIP overexpression in MBH reduces the energy expenditure and causes glucose intolerance, evidence that TXNIP regulates energy homeostasis with TRX in a binding-dependent manner [103]. MBH comprises the arcuate nucleus of the hypothalamus (ARH) and the ventromedial nucleus of the hypothalamus, which includes agouti related peptide (AgRP) neurons. Later studies revealed that AgRP neuron specific TXNIP deletions generated by AgRP-Ires-Cre mice and TXNIP flox/flox mice exhibit mild lean phenotype [104]. In addition to nutritional sensing, the hypoxic–ischemia or excessive ROS-dependent neuron toxicity under normal or diabetic conditions is caused by TXNIP induction [33,53,105,106,107,108]. Although these phenotypes are caused by the CNS, it does not explain the remarkable glucose tolerance and glucose disposable phenotype of TXNIP-WKO. CNS TXNIP may be an important player to control whole-body energy expenditure and adiposity. Recent evidence indicates that Parkinson’s disease and diabetes, both age and environmental stress-related chronic diseases, share remarkably similar dysregulation pathways. TXNIP was linked with dysregulation in β-cells, peripheral tissues for diabetes, and dopaminergic neurons, especially under high glucose conditions.

## 3. TXNIP/TBP-2 in Molecular Functions

### 3.1. TXNIP/TBP-2 as α-Arrestin, a Scaffold Protein Family

Although the TRX/TXNIP redoxisome signal complex is the primary concept of the molecular basis of TXNIP function [5], several remarkable features of TXNIP may provide the molecular mechanism of TXNIP in glucose homeostasis beyond redox signaling. Arrestins are protein families of scaffolding proteins for signal transduction in organisms. For example, β-Arrestins such as β-Arrestin 1 and β-Arrestin 2 bind to the G-protein coupled receptors (GPCRs) and desensitize them by multiple strategies, including preventing further activation and promoting receptor internalization and degradation [109,110,111,112]. In addition, β-Arrestins bind to Smoothened and Frizzeled receptors, the known seven-transmembrane-receptors (7TMRs) that mediate Hedgehog and Wnt signaling. β-Arrestin 2 is an important regulator of insulin signaling by direct binding to the insulin receptor (IR) [113]. Inteestingly, β-Arrestin 2 knockout (β-Arrestin 2KO) mice showed insulin resistance when eating a normal control diet. Insulin resistance occurs in WT mice by failing to recruit the thyrosine-protein kinase Src and the serin/threonine protein kinase Akt to the IR/β-Arrestin 2 signal complex [113]. Another Arrestin family is called as the α-Arrestins, which consist of ARRDC1-5 and TXNIP. ARRDC3 (which is originally identified as thioredoxin-binding-protein-2-like inducible membrane protein/TLIMP, the target of PPARγ [18]) acts as a membrane scaffold protein like β-Arrestins to regulate glucose uptake in adipose tissues [114]. TXNIP may exert its regulatory effects through its actions in the cytosol, nucleus, and mitochondria as well as intracellular matrix. It has been shown that TXNIP translocate to the intracellular matrix to promote the internalization of GLUT1 and restrict glucose uptake and glycolysis [115]. This novel TXNIP function at the cellular level was linked to the extracellular remodeling during tumorigenesis and embryogenesis. At tissue level, TXNIP regulates glucose homeostasis not only by regulating insulin signaling in adipose or muscles, but also by regulating gluconeogenesis in the liver, insulin secretion in the pancreatic β cells, and whole-body glucose disposals through CNS. These insights suggest that TXNIP is a more dynamic regulator of glucose homeostasis than β-Arrestins. TXNIP directly binds to importin α, a nuclear transportation protein providing the mechanism of nuclear shuttling from the cytosol of TXNIP [116] (Figure 2). GFP-tagged TXNIP overexpression or HDAC inhibitor SAHA-induced TXNIP induction revealed that TXNIP mainly localizes in the nucleus [116], suggesting that TXNIP may act as the scaffold protein in the nucleus for regulation of gene expression. We have shown that TXNIP suppresses PPARs signaling and activates SREBP signaling in the liver [11]. Protein purification aimed to identify TXNIP interacting protein in the rat β cell line INS-1 revealed that the candidate nuclear proteins binding to TXNIP include Mybbp1a, DEAD (Asp-Glu-Ala-Asp) box polypeptide 5 (Ddx5), GCN1, and NoO/p54nrb homolog [12]. More recently, it has been identified that TXNIP forms a high molecular weight complex (1000–1300 kDa) in redox sensitive manners [117,118]. This finding suggests that the scaffolding function of TXNIP may be modified by oxidative stress and the physical interaction with TRX. The shuttling of TXNIP to the mitochondria was found by H_2_O_2_ treatment in INS-1 cells and forms the protein complex with TRX2 (mitochondrial TRX) [85]. Further identification of protein complexes of TXNIP in different cell type with different physiological conditions may facilitate understanding of TXNIP’s molecular function.

### 3.2. Protein Degradation of TXNIP/TBP-2

The α-Arrestins family of proteins includes TXNIP and conserves the PPXY sequences that are known binding motifs of the WW domain [119,120,121]. Yeast Arrestin-related trafficking adaptors (ARTs), which are the homologs of mammalian ARRDCs, have been identified to contain PPXY motifs that interact with Nedd4-like ubiquitin ligase, Rsp5, resulting in ubiquitination and regulating the internalization of plasma membrane proteins (cargos) and degradation in the lysosome [121]. In humans, the NEDD family has nine members: NEDD4, NEDD4L, WWP1, WWP2, ITCH, SMURF1, SMURF2, HECW1, and HECW2 [120]. TXNIP undergoes proteasomal degradation by polyubiquitination through the physical interaction with the HECT ubiquitin ligase ITCH [122,123,124,125]. In contrast, TXNIP stabilizes p53 expression by interacting with human ecdysoneless (hEcd), which is known for its role in stabilizing p53 protein expression [126]. TRX stabilizes TXNIP protein expression, possibly preventing the interaction between the PPXY motif of TXNIP and the WW domain of NEDD ubiquitin ligase families [127] (Figure 2). There is growing evidence that TXNIP may act as the scaffold for proteins when it should be undergoing proteasomal degradation when not bound with TRX. TXNIP stabilizes protein expression when bound with TRX in response to a variety of stress signaling in a redox dependent manner.

## 4. TXNIP/TBP-2 in Clinical Work and the Future

**Figure 3:**
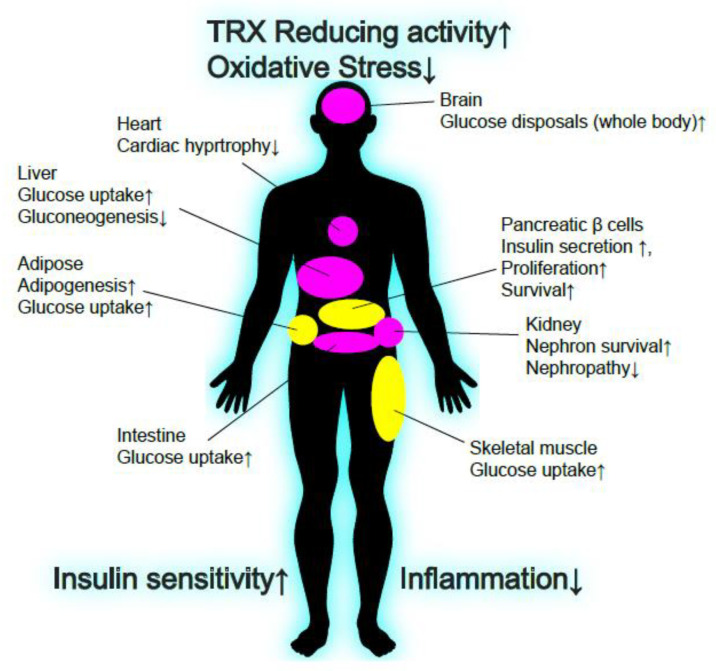
Beneficial effects of TXNIP inhibition for glucose homeostasis. Known effects of TXNIP down-regulation in glucose homeostasis are shown. TXNIP is known to be up-regulated in many tissues of a variety of pathogenic conditions, including T1D and T2D. Broadly, TXNIP down-regulation enhances TRX reducing activity and protects from oxidative stress. TXNIP down-regulation also enhances insulin sensitivity and suppresses inflammation. Tissue specific TXNIP down-regulation may provide safer treatment for T1D and T2D (yellow), and since TXNIP has anti-oncogenic functions in several tissues, chronic whole-body TXNIP inhibition may cause serious issues for cancer development (pink).

Given the remarkable function of TXNIP for glucose homeostasis, TXNIP has been recognized as an attractive target for treatment in both T1D and T2D. In addition, TXNIP possibly can be targeted to metabolic disease related complications such as cardio-vasculature disease [128,129,130,131] and kidney failure [132,133]. Verapamil was originally used for controlling ventricular rate in supraventricular tachycardia, migraine headache prevention, treatment of high blood pressure, and angina [134,135]. Recent studies revealed that Verapamil reduces TXNIP expression in multiple cell types in vivo and in vitro, including pancreatic β cells [76,136,137,138,139,140,141]. Since verapamil treatment in the multi low dose streptozotocin (MLD-STZ) induced mouse model of T1D and obese T2D model ob/ob mice preserved functional β cell mass and ameliorated hyperglycemia [76], a randomized-double blind, placebo controlled Phase 2 clinical trial with verapamil in adult subject recent-onset T1D was performed [140]. The study found once daily oral verapamil treatment for 12 month improves endogenous β cells insulin secretion function with a lower increase of insulin requirements and fewer hypoglycemic events in adult individuals with recent-onset T1D [140]. Interestingly, most recent study identified a small molecule that inhibit TXNIP expression and ameliorates hyperglycemia in both mice model of T2D (db/db) and T1D (STZ) [141]. It was shown that SRI-37330 treatments down-regulate TXNIP mRNA and protein level in rat β cell line as well as in mouse and human islets. The study also showed SRI-37330 reduces TXNIP-mediated glucagon secretion from α cells and suppress hepatic gluconeogenesis. Although the mechanism how SRI-37330 inhibits TXNIP expression, the specificity for targeting TXNIP and the molecular links between TXNIP and glucagon secretion is uncertain, these results encourage targeting TXNIP as promising anti-diabetic therapeutics (Figure 3). However, more tissue specific targeting of TXNIP is required for safer and efficient treatment of diabetes, since anti-oncogenic function of TXNIP has been well known [19,94,116,142,143,144,145,146,147,148,149,150,151,152,153], which suggests that chronic TXNIP inhibition in proliferative tissues such as the liver or intestine may increase the risk for tumorigenesis. Tissue specific physiological role of TXNIP give us a lesson to learn the targeting tissues for each specific condition in diabetes (Table 1). The specificity of TXNIP inhibition should be also considered since none of the known TXNIP modulators target only TXNIP, rather broadly affecting transcriptome [154]. In addition, although drug treatments such as insulin, glucagon-like peptide-1 (GLP-1/Exendin-4), thiazolidinediones (TZD), dipeptidyl peptidase 4 (DPP-IV) inhibitors, sodium–glucose co-transporter-2 (SGLT2) inhibitors, and metformin are successful to provide the therapeutics in T1D or T2D [155,156,157,158,159], none of these therapeutics provide a “functional cure” for the diabetes, which means life-long drug treatment to ameliorate diabetes is required for the patients. As a result, TXNIP targeting for diabetic therapeutics should be more specific rather than treatment with inhibitors to provide a functional cure for both T1D and T2D (Figure 3). Although, so far, the specific tissue or region targeting TXNIP therapeutics has not been demonstrated, one such idea is using gene delivery technology combined with genome engineering. Recent success of CRISPR associated protein 9 (Cas9) and targeted single guide RNA (sgRNA) delivery using adeno-associated virus (AAV) in selective regions of tissues in vivo provides evidence for the therapeutic utility of genome engineering technology [160,161]. Tissue specific TXNIP deletion in adipose or skeletal muscle exhibited powerful insulin sensitization with no evidence of tumorigenesis, therefore AAV-CRISPR mediated TXNIP deletion in those tissues would be beneficial for treating T2D. Using insulin promoter-driven Cas9 and TXNIP sgRNA expression in pancreatic β cell may also be effective to sustain the functional β cell mass in both T1D and T2D patients. Besides, direct gene editing of TXNIP *in vivo*, TXNIP modification in vitro may contribute for the advanced therapeutics in diabetes. For example, regulation of TXNIP expression may optimize the current protocol of human β-like cell generation from pluripotent stem cells. Although TXNIP-KO mice do not show any evidence for defects of β cell differentiation, TXNIP is one of the most highly responsive genes for high glucose and regulates both β cell function and survival. A recent study showed that in human pluripotent stem cells, derived insulin-producing β cells (sc-β cells) with or without gene collection of the pathogenic variant of Wolfram Syndrome (WS) revealed a 1.5–2-fold TXNIP gene induction by high glucose stimulation [162]. This is an encouraging observation; however, the induction rate of TXNIP genes in response to high glucose in human β cells lines or primary human islets are generally even higher (~10-fold) [12,72,73], suggesting that TXNIP signaling may not be fully activated in human pluripotent stem cell-derived insulin-producing β cells. Since TXNIP has been shown to regulate MAFA expression through miR-204 in murine and human islets [87], the current limited expression of MAFA in hPSC-derived insulin producing cells [89,163,164,165,166,167] might be potentially modified by TXNIP expression. Glucose responsiveness of TXNIP gene expression may provide the fine tuning of glucose responsiveness of human pluripotent stem cell-derived insulin-producing β cells.

## 5. Conclusions

After the discovery that TXNIP/TBP-2 is a binding partner of the antioxidant TRX, the TRX/TXNIP signal complex in redox-dependent (redoxisome) and -independent pathways has been studied. Current emerging evidence clearly suggests that TXNIP is a central master regulator of whole-body glucose homeostasis in both rodents and humans. To further facilitate therapeutics to provide a functional cure for diabetes, more sophisticated TXNIP-targeting therapeutics should be developed using state-of-the-art biotechnology. We still do not fully understand the basic molecular mechanisms of how TXNIP interacts with other proteins, responding various stimuli in the different cell types and different cellular localizations. Of great interest, probably TXNIP function to inhibit TRX activity by direct binding is important not only for their redox sensitive regulation, but also for their redox independent function thorough structural changes of protein complex. Further investigation of redox-dependent and -independent scaffolding functions of TXNIP may give us deeper insights of the molecular functions of TXNIP in pathophysiology and future therapeutics for diabetes.

## Figures and Tables

**Table 1:** Physiological role of TXNIP in normal, obese, STZ, endotoxin, ischemia, and diabetic conditions. The key physiological findings based on genetic knockout (KO) or mutated mice are summarized. ↑ Up-regulation/Enhanced function, ↓ Down-regulation/Reduced function.

Tissue/Cell Type	TXNIP Function	Key Signal	TXNIP Whole Body KO/Mutant	TXNIP Tissue Specific KO/Mutant	Reference
β cells (Normal)	Apoptosis↑ Glucose-stimulated insulin secretion (GSIS)↓	Mitochondria metabolism TRX/TRX2/ROS MAFA/miR-204 IAPP/miR-24a Zeb1/miR-200	GSIS↑ Hyperinsulinemia↑	β cell mass↑ Apoptosis↓	[11,12,41,86,87,88]
β cells (STZ/Obese/Diabetic)	Apoptosis↑ Glucose-stimulated insulin secretion (GSIS)↓ β cells hypertrophy↓	Mitochondria metabolism/uncoupling AKT/Bcl-2 UPR/ER stress	GSIS↑ Hyperinsulinemia↑ Apoptosis↓	Hyperinsulinemia↑ β cell mass↑ Apoptosis↓	[12,13,14,78,79]
Skeletal muscle (Normal)	Insulin sensitivity/Glucose uptake↓	AKT/GLUT4 PTEN AMPK	Insulin Sensitivity↑	Insulin Sensitivity↑	[12,16,32,91,130]
Skeletal muscle (Obese/Diabetic)	Insulin sensitivity/Glucose uptake↓ (Insulin Resistance↑)	AKT/GLUT4 PTEN	Insulin Sensitivity↑		[12,16]
Adipose (Normal)	Insulin sensitivity/Glucose uptake, Adipogenesis↓	AKT/GLUT4	Insulin Sensitivity↑		[11,12,16,32]
Adipose (Obese/Diabetic)	Insulin sensitivity/Glucose uptake↓ (Insulin Resistance↑)	AKT/GLUT4	Insulin Sensitivity↑	Insulin Sensitivity↑	[12,15]
Liver (Normal)	Gluconeogenesis↑ Lipogenesis↓	FOXO1 SREBP PPARα AKT	Abnormal steatosis in fasting	Normal glycemica, Hypoglycemia in fasting	[7,8,9,10,11,16,43,98,99]
Liver (Obese/Diabetic)	Gluconeogenesis↑ Lipogenesis↓	PRMT1/PGC1a	Lipogenesis↓		[12]
Immune cells (Normal)	Inflammation↓ Tumor rejection↑ Hematopoetic Stem Cells↓	NLRP3 inflammasome TRX/ROS P38	NK cells↓ T-cell response↓		[44,45,46,47,48]
Immune cells (Obese/Diabetic/Endotoxin)	Inflammation↓	PI3K/ROS NO	Resistant to P. aeruginosa-induced bacteremic shock Metabolic disordering by LPS		[46,47]
Brain (Normal)	Glucose uptake↓	TRX/ROS AKT/GLUT4	Glucose uptake↑		[32]
Brain (Obese/Diabetic)	Energy expenditure↑ Adipogenesis↓ Body Weight↑ Insulin resistance↑	TRX/ROS		Hypothalamus:Body Weight↓ Insulin resistance↓ AgRP Neuron: Energy expenditure↓ Adipogenesis↓	[103,104,105]
Heart (Normal)	Fatty Acid oxidation↓ Glucose Oxidatation↑	miR33/AMPKα		Fatty Acid oxidation↑ Glucose Oxidatation↓	[131]
Heart (Obese/Diabetic/Ischemia)	Mitochondria↑		Resistant for ischemia-reperfusion injury		[128,129,130]

## Abbreviations

VDUP1: Vitamin D3 Upregulated protein; TBP-2, Thioredoxin Binding Protein-2, TXNIP, Thioredoxin-interacting Protein; TRX, Thioredoxin; NRs, nuclear receptors; PPARs, peroxisome-proliferator activated receptors; GR, glucocorticoid receptor, VDR, vitamin D receptor; FXR, farnesoid X receptor; TRX2, Thioredoxin-2; ASK1, Apoptosis signal regulating kinase 1; ChREBP, Carbohydrate response element binding protein; NLRP3, Nod-like receptor protein 3; ROS, Reactive oxygen species; T1D, Type-1 diabetes; T2D, Type-2 diabetes.
